# Aligning vertical interventions to health systems: a case study of the HIV monitoring and evaluation system in South Africa

**DOI:** 10.1186/1478-4505-10-2

**Published:** 2012-01-26

**Authors:** Mary Kawonga, Duane Blaauw, Sharon Fonn

**Affiliations:** 1 School of Public Health, Faculty of Health Sciences, University of the Witwatersrand, 7 York Road, Parktown, 2193. South Africa; 2 Centre for Health Policy, School of Public Health, Faculty of Health Sciences, University of the Witwatersrand, P.O Box 1038, Johannesburg 2000. South Africa

**Keywords:** integration, vertical programme, monitoring and evaluation, HIV, health systems

## Abstract

**Background:**

Like many low- and middle-income countries, South Africa established a dedicated HIV monitoring and evaluation (M&E) system to track the national response to HIV/AIDS. Its implementation in the public health sector has however not been assessed. Since responsibility for health services management lies at the district (sub-national) level, this study aimed to assess the extent to which the HIV M&E system is integrated with the overall health system M&E function at district level. This study describes implementation of the HIV M&E system, determines the extent to which it is integrated with the district health information system (DHIS), and evaluates factors influencing HIV M&E integration.

**Methods:**

The study was conducted in one health district in South Africa. Data were collected through key informant interviews with programme and health facility managers and review of M&E records at health facilities providing HIV services. Data analysis assessed the extent to which processes for HIV data collection, collation, analysis and reporting were integrated with the DHIS.

**Results:**

The HIV M&E system is top-down, over-sized, and captures a significant amount of energy and resources to primarily generate antiretroviral treatment (ART) indicators. Processes for producing HIV prevention indicators are integrated with the DHIS. However processes for the production of HIV treatment indicators by-pass the DHIS and ART indicators are not disseminated to district health managers. Specific reporting requirements linked to ear-marked funding, politically-driven imperatives, and mistrust of DHIS capacity are key drivers of this silo approach.

**Conclusions:**

Parallel systems that bypass the DHIS represent a missed opportunity to strengthen system-wide M&E capacity. Integrating HIV M&E (staff, systems and process) into the health system M&E function would mobilise ear-marked HIV funding towards improving DHIS capacity to produce quality and timely HIV indicators that would benefit both programme and health system M&E functions. This offers a practical way of maximising programme-system synergies and translating the health system strengthening intents of existing HIV policies into tangible action.

## Background

The purpose of monitoring and evaluation (M&E) is to produce reliable and timely health information and use it to evaluate policy, set priorities, plan, and monitor the effectiveness and impacts of interventions [1,2]. In recent years, many low- and middle-income countries have established dedicated (or vertical) M&E systems for their HIV programmes [3,4]. The anticipated aims of such M&E systems have however not been realised in many countries due to low financial investment in M&E infrastructure, weak or ill-defined systems for collection, analysis, and dissemination of HIV data, inadequately trained data collectors, and insufficient technical capacity to transform HIV data into usable indicators [3,4]. The non-integration of HIV M&E systems with overall health information systems is another important factor. Vertical M&E systems are often coordinated as separate parallel systems, often in a bid to improve the availability of quality HIV information for decision-makers. However, this intended benefit is often not realised [3-7].

South Africa has established a vertical HIV M&E system to monitor its national HIV programme [8]. In South Africa's decentralised health sector, the district (sub-national) level of the health system is well-placed to use information generated by this HIV M&E system to monitor the HIV programme. However, it has not been documented if and how the HIV M&E system interacts with the district health information system (DHIS) designed to monitor overall health system performance at district level, or whether it affects the availability of HIV programme information at district level. This paper addresses this gap.

### Disease-specific programmes and health systems

The emergence of global health initiatives (GHI)-notably the Global Fund for AIDS, TB and Malaria (GFATM) and the Presidential Emergency Plan for AIDS Relief (PEPFAR)-as major funders of HIV/AIDS interventions in low- and middle-income countries-has raised questions about the sustainability of disease-specific (vertical or targeted) programmes [9,10]. GHIs increase HIV funding and services [11], but also fragment coordination by establishing parallel planning, coordination and monitoring systems within recipient countries, and worsen already weak health systems by diverting resources from general health services [12,13]. This has prompted calls to strengthen health systems and find ways of maximising positive synergies between disease-specific programmes and health systems [9,14].

Integration of disease-specific programmes into health systems is one way of strengthening health systems and maximising programme-system synergies [15]. Integration is largely understood in relation to the service delivery function of health systems: e.g. combining two or more disease-specific services at one delivery point, incorporating disease-specific services into general care, continuity of care over time or across levels, or working across government sectors [16-19]. Health system impacts of service integration are however inconclusive due to poor evidence because of weak or incomparable evaluation methodologies [18-20]. There is even less evidence on how targeted programmes interact with health system functions other than service delivery, such as financing, M&E, or governance [13,18]. In practice programmes lie along a continuum from integrated to fully vertical, and depending on the context, integrate with different health system functions to varying extents [10,13]. In the absence of conclusive evidence on effects of integration, it is advised that countries adopt organisational arrangements that optimise benefits for both programmes and systems in their context-specific settings [9].

The high cost of maintaining parallel disease-specific programmes, and the potential benefits of integrated services with unified finance, management and M&E processes provide compelling reasons to adopt organisational arrangements that optimise programme integration, particularly in weak health system contexts [15]. Some even suggest there are very few instances when integration should not be the norm [21]. Countries however need guidance on when and how to integrate disease-specific programmes to strengthen health systems. For example, in South Africa health system strengthening is a stated HIV programme goal [22,23] but how to achieve this is unclear. Documenting how the HIV programme interacts with and affects health system functions is a step towards clarity.

### HIV programme and the health system in South Africa

South Africa's post-apartheid HIV programme was established in 1994, initially emphasising prevention. Public sector antiretroviral therapy (ART) services were introduced in 2004 with earmarked funding following the Operational Plan for Comprehensive HIV/AIDS Care Management and Treatment (comprehensive plan) [22,24]. An HIV M&E system was established to monitor the comprehensive plan [8,22], and after adoption of the multi-sectoral HIV & AIDS and STI Strategic Plan 2007-2011 (NSP), the M&E framework was expanded to include other sectors [23].

Health services in South Africa are largely funded through the National Treasury. External funding constitutes less than 1% of the National Department of Health (NDOH) budget [25-27]. However, South Africa does receive GHI funding (largely from GFATM [dispersed to Government] and PEPFAR [dispersed to non-governmental agencies]), the bulk of which goes to HIV/AIDS. As such, external aid constitutes 26% of the government's HIV/AIDS budget [25].

The National Treasury funds health largely through an annual unconditional block grant named the 'equitable share' (based on population numbers and needs) which is allocated to provincial governments, who then distribute this between various departments including health. In addition, the National Treasury allocates to each provincial government dedicated funding for HIV-this is termed the conditional grant for HIV and AIDS [27]. Reporting on expenditure for conditional grants is different to that required for equitable share funding. The Division of Revenue Act (DORA) provides the legislative and accountability framework for the HIV and AIDS conditional grant, requiring provinces to submit HIV data as well as narrative and financial reports to the National Treasury [28].

Health system decentralisation has also been a national priority since 1994. As such, responsibility for managing health service delivery is decentralised to the district (sub-national) level [29,30]. A district health information system (DHIS) has been established to support district health management teams (DHMT) in this role [31,32]. The DHIS-a critical component of the national health information system-collects public sector facility data to produce a set of district health service indicators. Ideally, the disease-specific HIV M&E system should be a sub-component of a system-wide information system like the DHIS [1]. As such, DHMTs would be well-placed to integrate HIV information into overall district health system management.

A national study however reports that programme information generated through disease-specific M&E systems is not necessarily made available to district managers [33]. Though that report did not specify the HIV M&E system, it highlights the need to understand how the HIV M&E interacts with the health system M&E function (the DHIS) to determine whether any negative synergies between them affect the availability and use of HIV information for district management. This paper thus uses a district in South Africa as a case study to determine the extent to which the HIV M&E is integrated with the DHIS, and assess effects of the HIV M&E on availability of HIV information at district level. The paper also discusses factors influencing HIV M&E integration, and proposes ideas for maximising HIV programme-system synergies.

## Methods

This cross-sectional descriptive study was conducted during April to July 2009 in one of South Africa's nine provinces. This largely rural province is divided into 3 district municipalities (districts). Districts are further split into several local municipalities (sub-districts) and overseen by district and sub-district managers, respectively. Each sub-district has several service delivery local areas (clusters of clinics), each overseen by a local area manager (or clinic supervisor). A manager at provincial level oversees the HIV programme assisted by four deputies-one each for prevention, care and support, treatment, and M&E sub-programmes-supported by assistant managers at provincial level and HIV programme managers at district and sub-district levels.

The prevention sub-programme in the study site included condom distribution, life skills and behaviour change, VCT, PMTCT; and later post-exposure prophylaxis for rape survivors (PEP) and HIV/TB collaboration (management for HIV-TB co-infected people) were added. At the time of the study the PMTCT protocol had just changed from single dose nevirapine (NVP) to short-course NVP and zidovudine (AZT). Prevention services were largely funded through equitable share funding. The treatment sub-programme focused on ART services whereby accredited comprehensive care, management and treatment [CCMT] sites initiated patients on ART, and down-referral sites managed those already initiated and stabilized on ART. During the study, there were 32 CCMT sites in the province (23 at hospitals, 9 at community health centres, and none at clinics). CCMT services were funded through the HIV conditional grant. About 75% of the province's conditional grant was allocated to CCMT services.

We purposively selected one of the three district municipalities in the province (one of 46 district municipalities in the country) for practical reasons as we were already working in that site. Like other district municipalities, our study site used the national DHIS and was also required to report to national level on a set of nationally-defined HIV indicators. We identified the most functional of five sub-districts in our study district, and within this, selected both CCMT sites and both down-referral sites, and used stratified sampling to select one clinic from each of seven local areas (the strata) (Table 1). Only one of our selected health facilities (a CCMT site) was NGO supported and thus reported HIV data to its funders as well as government. All our other facilities were fully government-funded and did not report HIV data to external donors.

**Table 1:** Study sample and participants

Type of facility	Type of HIV service	No. in sample	Data collection tools
Hospital*	HIV treatment (ART site)	2	Facility checklist
Community health centre^@^	HIV prevention** HIV treatment (DR site)^#^	2	Facility checklist
Primary care clinic	HIV prevention	7	Facility checklist

**Interview participant**	**Level of health system**	**No. in sample**	**Data collection tools**

Facility (operational) manager	Health facility	11	Semi-structured questionnaire
HIV programme manager	Provincial	4	In-depth interview guide

*These are dedicated HIV treatment clinics located within the hospital

**HIV prevention services include VCT, PMTCT, TB/HIV collaboration services, and PEP

^@^Like clinics, community health centres (CHC) are nurse-driven health facilities providing primary care services. Clinics offer an 8-hour daily service; CHCs offer a 24 hour services, including maternity delivery services.

^#^DR = down-referral ART site

We interviewed four senior HIV programme managers at provincial level and the operational managers of all 11 facilities where we also reviewed M&E documents. Ethical approval was granted by the University of the Witwatersrand and the Provincial Department of Health. Interviews were in English, tape-recorded where consented, transcribed, and analysed thematically.

The variables we measured are outlined in Table 2. To describe the HIV M&E system design we assessed whether it possessed the organisational attributes required of a national M&E system including: a comprehensive M&E plan with clear goals, targets and resources for its implementation, clear plans for data collection and analysis of defined indicators, and an appropriately staffed M&E unit to coordinate activities [4,34]. To examine integration we adopted the methodological approach of Atun et al., [35] which measures the extent to which governance, planning, service delivery, demand generation, financing and M&E functions of vertical programmes are integrated with those of the health system. We assessed only the M&E function and examined the following M&E activities: data collection, collation and reporting, and analysis. Guided by definitions of integration as shared resources and technologies [36] and shared or merged coordination responsibility [21,35], we measured the extent of integration as "full integration" if M&E activities and resources and coordination were shared between the HIV M&E system and the DHIS, "partial" if there was some, and "no integration" if there was no sharing. We measured availability of HIV indicators as the outcome of interest. Although information use is the ultimate purpose of an M&E system [1,7], availability can be a marker of M&E system performance [4].

**Table 2:** Variables measured in the study

Variables	Definition
Design of the HIV M&E*	▪ Existence of an M&E framework and plan ▪ Definition of data elements/HIV indicators ▪ Availability of financial resources ▪ Existence and staffing of M&E unit

Activities for the production of HIV information	▪ *Collection*: number of data recording forms in use; number and purpose of HIV data elements recorded. ▪ *Collation and reporting*: number of data collation forms; mechanisms to transmit data from facility to higher levels of health system, format for reporting and audience. ▪ *Analysis*: approach to HIV data analysis at different levels of the health system.

Availability of HIV information	▪ Whether HIV indicators are disseminated to and available at the district level.

Extent of HIV M&E and DHIS integration	Extent of sharing of: ▪ *Collection*: personnel and forms that record HIV and DHIS data. ▪ *Collation and reporting*: personnel and forms that collate HIV and DHIS data; reporting pathways and mechanisms, and audience to whom HIV and DHIS indicators are sent. ▪ *Analysis*: shared personnel and analytic approach for HIV and DHIS data.

*This variable measures whether the required organizational attributes of an M&E system are in place [4,34].

## Results

### Design of the HIV M&E system

We found that the HIV M&E was designed at national level, in accord with the national HIV programme M&E framework. The national level defined what HIV data to collect and report (these changed as services were modified or added) and designed the data recording and collation forms. Respondents reported that conditional grant funding was used to establish an HIV M&E unit at provincial level and to appoint an M&E unit manager and a data clerk at each ART (CCMT) site to specifically coordinate the production of ART indicators. The production of HIV prevention information was coordinated by the provincial health information unit which managed the DHIS. This unit was staffed by an information manager and a health information officer, and supported by one information officer and one data clerk each at district and sub-district levels, respectively.

### The production of HIV information

Our data showed that HIV prevention data were manually recorded on four different tick registers which were in use at most clinics and all down referral sites (Table 3). HIV treatment data were manually recorded on six different forms including an ARV monitoring register to record individual patient data at monthly follow-up visits, and five other forms, two of which respondents referred to as "DORA reporting tools" (Table 4). As shown in Table 4, the ARV monitoring register was in use at both down-referral sites (completed by nurses) and both CCMT sites (completed by data clerks). Informants at both down-referral sites and one CCMT site reported that staff did not use the other five ART data recording forms because they found them to be too complicated to complete.

**Table 3:** Use of nationally-designed forms for recording HIV prevention data

Type of form	Description of form	Use of form at primary care clinics (n = 7)		Use of form at DR sites (n = 2)	
	All forms were tick registers	No. of clinics using form	Health worker completing the form^#^	No. of DR sites using form	Health worker completing the form^#^
PHC (HIV) register*	Records data on VCT, PMTCT, CD4 test before ART, management of TB/HIV co-infected patients, referrals to ART service.	6	Lay counsellor	2	HIV nurse

VCT register**	Records data on HIV counselling, HIV testing, and HIV result for different client types (pregnant women, TB patients, others).	7	Lay counsellor	2	Lay counsellor

Antenatal NVP register	Records data on Nevirapine (NVP) dispensed to HIV positive pregnant women for PMTCT.	6	PHC nurse	2	Maternity nurse

Antenatal PMTCT register***	Records data on Zidovudine (AZT) dispensed to HIV positive pregnant women for PMTCT.	5	PHC nurse	2	PHC nurse

^#^In both clinics and DR sites, PHC nurses (provide general care) also complete the DHIS register. 'HIV nurses' did not use the DHIS form.

*The PHC (HIV) register was introduced in early 2009, and a revised version was introduced in June 2009.

** The VCT data that this form collects are also recorded on the PHC (HIV) register.

*** This form was introduced in May 2009 (2 months before data collection) after inception of dual therapy for PMTCT.

**Table 4:** Use of nationally-designed forms for recording HIV treatment (ART) data

Name of form or register	Brief description of form	No. of ART (CCMT) sites using this form (n = 2)	No. of ART (DR) sites using this form (n = 2)
**For recording HIV treatment (ART) data-for M&E reporting**			

ARV patient treatment monitoring register	This records individual patient data-ART regimen, CD4 and viral load levels, and patient weight. Data from this register are tallied on the ARV patient M&E and DORA tally sheet (described below).	2	2
ARV patient M&E data elements collection tally sheet	This sheet tallies-by age, sex, and pregnancy status-No. eligible for ART, type of ART regimen. It records No. of activities and not individuals.	0	0
ARV laboratory data collection tally sheet	This sheet tallies number of CD4 count and viral load tests done-those at staging visits (baseline) and at 6-month follow-up visits. Data are not patient-linked.	1	0
ARV patient nutritional supplements tool	This form records the number of various nutritional supplements disbursed to HIV + patients, weight, and No. of deaths.	0	0

**For recording HIV treatment (ART) data-DORA reporting**			

ARV patient DORA data elements collection tally sheet	This form tallies the No. Of patients assessed for ART, commenced on ART, deregistered, and adherent to ART. It also records clinical and biological monitoring of ART patients. Data are activities and not patients.	0	0
Daily stock-out control tool	This form records the number of days nutritional supplements and specified drugs are out of stock in any given month.	1	0

In total 201 HIV data elements were recorded, though some were duplicated-e.g. the same VCT data was recorded on two different forms (Table 5). A review of data forms for June 2009 revealed that while VCT registers at all clinics and down-referral sites and the ARV patient monitoring register at one CCMT site were complete, all other forms had missing fields or were not used at all. Facility managers attributed the incomplete recording and non-use of forms to their staff not being trained, particularly on newly-introduced data elements and registers.

**Table 5:** Number of HIV data elements collected with nationally-defined forms

Aspect of HIV service and type of form used to record data	No. of data elements recorded
**HIV prevention data: recorded on the PHC (HIV) register**	**44**

VCT	9
PMTCT	12
TB/HIV collaboration services	7
Post-exposure prophylaxis for rape survivors	8
Assessment for ART	7
STI treatment in ART patients	1

**HIV prevention data: recorded on the VCT register**	**11**

VCT service use	11

**HIV prevention data-recorded on PMTCT registers**	**11**

Nevirapine dispensed to pregnant women	4
AZT dispensed to pregnant women	7

**HIV treatment data-recorded on ART register and tally sheets**	**135**

ART assessment-for ART eligibility and drug readiness	25
ART follow-up-clinical, laboratory, drug regimen (register)	9
Viral load and CD4 testing-at baseline and follow-up (tally)	36
Dispensing of nutritional supplements (tally)	22
ART services-tally of activities for ART assessment and follow-up	33
Stock control-tally of stock outs in previous month	10

Staff at clinics and down-referral sites designed and used their own additional HIV forms (Table 6). For example, some designed registers to track patients who were eligible for ART, which the nationally-defined forms did not appear to enable them to do. Neither CCMT site implemented additional facility-specific forms, but respondents reportedly observed this practice at other CCMT sites in the province:

"Initially when the program started, they sort of used note books before we could introduce this tool. So it is difficult for them to change from their note books to the tools; they think the [DORA] tools are a bit too complicated. They stick to their note books. But you will find one facility having more than five or eight note books-so they are still sticking to their note books, because initially they have been made to improvise to use note books, so is not easy for them to change".(programme manager)

**Table 6:** Facility-specific forms for recording HIV data

Types of data collected with facility-specific forms*	No. of clinics (n = 7)	No. of down-referral sites (n = 2)
VCT service use	5	1
CD4 testing and results by patient name	4	2
TB/HIV prevention (collaboration)	-	1
Pre-ART support, ART readiness, referral to CCMT site for ART	6	2
Down-referrals from ART site	N/A	2

**No. of facility-specific forms in use per facility**		

No. of facilities using 1 facility-specific form	1	-
No. of facilities using 2-3 forms	6	-
No. of facilities using > 3 forms	0	2

*None of the CCMT sites developed facility-specific data recording forms

**Figure 1:**
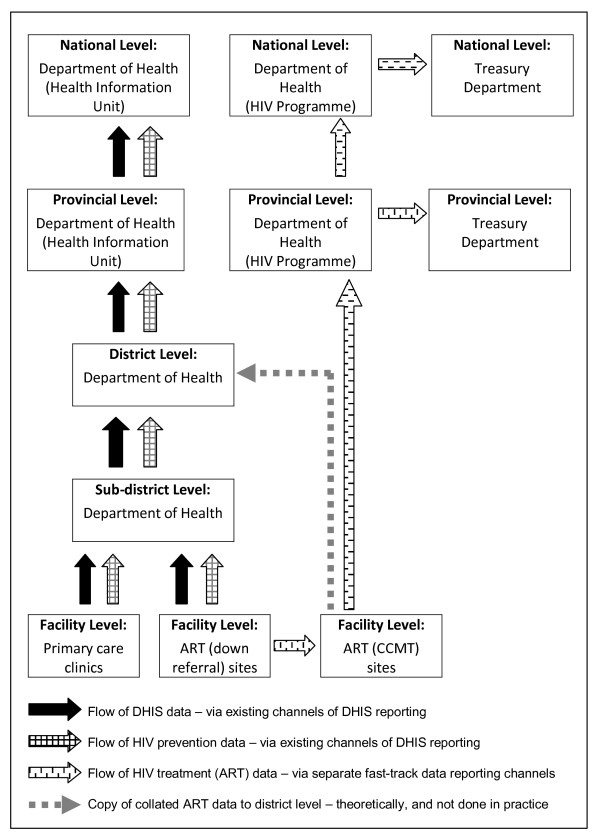
**flow of HIV and DHIS data through various levels of the health system**. The flow of HIV data and DHIS data from the health facility level (where data are recorded) to the national level of the health system was described, based on the data obtained through key informant interviews and review of M&E documents at facility level. The figure indicates that the flow of HIV treatment data is separate from and parallel with the flow of DHIS data.

HIV prevention data that were recorded on VCT and PMTCT registers were manually aggregated by senior nurses on a DHIS monthly collation form and submitted to the sub-district level where the information officer captured the data on DHIS software and transmitted it electronically to the district and then the provincial health information unit. Analysis at sub-district and district level generated DHIS and HIV prevention indicators. ART data were collated on a monthly ARV collation form by data clerks at both CCMT sites (the two DR sites sent their data to CCMTs site for collation) and submitted directly to the HIV M&E unit where the HIV M&E manager captured and aggregated the data as counts and no analyses were done (Figure 1).

### Availability of HIV indicators at district level

From the provincial level, HIV data were submitted to the National Health Department (prevention and ART data) and the National Treasury Department (ART data) (Figure 1). The latter was referred to as "DORA reporting". The HIV M&E manager compiled the DORA report which included ART data and a few VCT and PMTCT indicators (supplied by the provincial information unit). HIV prevention data were available at the district level (as they were incorporated into the DHIS) and included in the district quarterly review (DQR)-a process where district health managers use health service data to review district performance. CCMT data were shared amongst HIV programme managers at provincial and district level but were not incorporated in the DHIS and not included in the DQR process.

### Integration of the HIV M&E with the health system M&E function

Overall, the prevention sub-programme was partially integrated with the DHIS-data collection activities were partially integrated while the rest were fully integrated-while the treatment (ART) sub-programme M&E was not integrated with the DHIS (Table 7). 

**Table 7:** Extent of integration of the HIV M&E system with the DHIS

M&E activity	HIV prevention sub-programme	HIV treatment sub-programme
	*Overall: partial integration*	*Overall: no integration*
**Data collection**	*partial integration*	*no integration*

Data recording forms	Forms for HIV prevention data are separate from DHIS forms.	Forms for ART data are separate from DHIS forms.
Personnel who record the data	Dedicated HIV personnel record most prevention data; some sharing only at clinics where PHC nurses record some PMTCT and all DHIS data.	Dedicated CCMT data clerks capture only ART data.

**Data collation and reporting**	*full integration*	*no integration*

Data collation forms	Data are collated on the standard monthly collation form that is used for DHIS data.	DORA monthly collation form-separate from DHIS data collation form.
Data reporting (also see Figure 1)	Data reported through DHIS reporting mechanisms.	Data reported through dedicated channels separate from the DHIS.

**Data analysis**	*full integration*	*no integration*

Approach and level at which data analysed	Same analysis approach as DHIS; analysis at sub-district and district levels by same staff who analyse DHIS data.	DORA reports analysed at national level-separate analysis from DHIS.

**Dissemination**	*full integration*	*no integration*

Information dissemination processes	Indicators disseminated through the DHIS to managers at district and provincial levels.	DORA reports disseminated to the National Treasury and National Departments of Health-not through the DHIS.

Respondents reported that ART data were coordinated separately to ensure DORA reporting deadlines were met, and because there was little confidence in the DHIS, as illustrated below:

"Because the CCMT data has got this political pressure of saying that by the 10th we are supposed to report at the National Department of Health. That is why by the 3rd they are supposed to be faxing directly. If we say they should report at the sub-district level then data is going to take a month to be at the provincial office, of which it is unacceptable." (programme manager)

"And another thing with the data that we are getting from the CCMT sites, ideally one would be even more happy if we were getting that data through the normal health facility data reporting, through the district health system. But it is not falling in the district health system; it goes directly from the facility to the province. And the only reason for that ...it's because, it says we are not confident with the data flow system and if we were to follow that data flow system as we should, probably we would be having nothing." (programme manager)

## Discussion

The HIV M&E system described in this study essentially meets the organisational requirements of a national M&E system, but has several limitations that affect its efficiency and utility and constrain the availability of HIV data. Below we discuss these limitations and factors that may influence HIV M&E integration in South Africa. Finally, we use our case study findings to propose some ideas about how integration can strengthen health systems and maximise programme-system synergies.

### Limitations of the HIV M&E system

The M&E system is designed and implemented in a top-down uncoordinated manner. It is characterised by a massive data set, duplication of data collection, incomplete data recording, and non-use of nationally-defined forms. These features have been shown to limit the efficiency and accuracy of HIV M&E systems in other settings [4,37]. Our findings indicate these problems exist because the HIV M&E was seemingly designed in an uncoordinated and top-down and manner-some data were collected but not collated and analysed, data forms were introduced without ensuring they did not duplicate existing ones or increase administrative burdens, and health workers' perceptions of the forms were not considered.

Another feature of the uncoordinated design is that the M&E system (particularly the ART component) largely generates data on service quantity (e.g. the number of people on ART) rather than service quality and outcomes. This skewed focus could be because service quantity data are needed for conditional grant reporting, but is limited as a good M&E system should also generate patient outcome indicators to monitor programme performance [1] and service quantity data are not sufficient to monitor performance. The limited utility of the HIV M&E is underscored by a recent report that despite the large amount of data gathering activity, the HIV programme has insufficient information to guide action [38]; and research which shows that routinely collected public sector ART service data do not allow monitoring of key performance measures like death, loss to follow-up and retention in care [39].

A further limitation of the HIV M&E system is that its vertical design contradicts the notion of integrated management. While M&E activities for the production of HIV prevention data are integrated with the DHIS, those for the production of HIV treatment data are not, which creates silos within a silo-i.e. within the vertical HIV programme, parallel M&E processes for prevention and treatment data. This silo approach is not unique to South Africa. In other settings distinct M&E systems for different components of the HIV programme exist, with health facilities reporting separately on each component [3]. In our study, the silo M&E approach could potentially promote divisions in the management of HIV sub-programmes, which contradicts stated goals of an integrated HIV/AIDS response [23]. The silo approach also limits the availability-and potential use-of ART information at district level because ART data by-pass the DHIS, and undermines national policy intents of integrated district management [29,30].

### Factors influencing integration of the HIV M&E system

A recent systematic review of the evidence on integration of disease-specific programmes into health systems reveals a dearth of analyses of the information (M&E) building block. That review also reveals a "highly heterogeneous picture" with programmes integrating with different health system functions to varying extents-though the dearth of cross-site comparable data precludes firm conclusions about which factors impede and promote integration [13]. The evidence however suggests that context-specific factors related to health system characteristics (e.g. fragility, absorptive capacity) and the politico-economic and socio-cultural context (e.g. commitment of national leadership, health personnel preferences) influence the extent of integration [13]. Based on our study, we propose that the following politico-economic and health system factors may influence integration of the HIV M&E system.

#### Politically-driven ART exceptionalism in a post-AIDS denialism era

Initially conceptualized as an integrated programme in line with national policies, the HIV programme was however introduction as a targeted intervention with special access to ear-marked funding [40,41]. This AIDS exceptionalism-treating AIDS as different from other public health threats [42], was fuelled by fears of health system incapacity and pressure to quickly curb a growing HIV epidemic [40,41]. At its inception the HIV programme however focussed only prevention activities, and HIV treatment was introduced much later, following pressure and legal action from civil society actors [43]. Government's delayed action on ART provision, largely attributed to the then-president Mbeki's AIDS denialism [43], resulted in a huge unmet need for ART in South Africa [44].

The introduction of public sector ART provision in 2004, hailed as an essential step forward, was however accompanied by a significant shift in focus from HIV prevention to treatment. This ART exceptionalism (prioritising ART above other HIV control foci)-as evidenced by the preferential funding and resources for ART M&E observed in this study-was seemingly a bid to meet new ambitious ART coverage targets to address the huge backlog. ART prioritisation has been heightened further following the launch in April 2010 of a presidential HIV counselling and testing (HCT) campaign to test 15 million sexually-active adults for HIV by June 2011, and new guidelines for earlier ART initiation of pregnant women, TB patients and children [45,46]. These initiatives have accelerated country-wide accreditation of many primary care facilities (clinics and community health centres) as CCMT sites [47]. In our study province, accrediting all primary care facilities would lead to a 10-fold increase in the number of CCMT sites, which raises questions about the long-term sustainability of a non-integrated ART M&E.

#### Ear-marked HIV funding

The desire for control over their funded programme often drives donors to continue supporting even inappropriate vertical systems [21]. Research shows GHI-supported systems often satisfy donor interests, and undermine recipient priorities, often contradicting donors' stated aims to harmonise in-country coordination [12,48,49]. Like many donors, National Treasury reporting requirements-reflecting a desire to closely monitor the conditional grant-facilitate an M&E system that does not primarily serve the interests of recipient provinces and is at odds with the notion of integrated M&E. While the conditional grant provides a legal mechanism for government to ensure provinces spend on national priorities [50], its reporting mechanisms are at odds with the DORA legislation through which it is administered. The DORA states that systems for monitoring the conditional grant should not impose an "undue administrative burden" on recipients and "should be compatible and integrated with" other systems [51].

#### Lack of confidence in the capacity of the national health information system

Respondents in our study expressed concerns about the capacity of the DHIS to manage and ensure timely reporting of data. DHIS weaknesses reported in the literature include poor quality data, incomplete reporting, delayed availability of information, low data use, and inadequate information management skills of DHIS personnel [31,52,53]. The existence of weaknesses should however not justify a silo M&E approach whereby ART data by-passes the DHIS to fast-track reporting to the national level. Using DHIS weakness to justify non-integration of M&E systems sends an incorrect message that the primary purpose of M&E is to submit data 'upwards' to higher levels for their use when in fact the message should be that M&E systems must principally support sub-national levels (districts) as the primary users of information. It is also a missed opportunity to strengthen the capacity of the DHIS to oversee the production of HIV information and ensure its use within the overall health system M&E function.

### Proposals for integration to maximise positive programme-system synergies

Maximising positive synergies between programmes and health systems is about finding practical ways in which disease-specific and health system functions can positively interact for optimal health benefit [54]. Below we highlight some ideas of what this may mean at sub-national (district) level, using our South African case study as an example.

The vertical HIV M&E system observed in this case study promotes fragmented health system coordination and seemingly benefits neither the HIV programme nor the health system. For HIV M&E systems set up like this we propose integration with the overall health information system (HIS), while strengthening HIS capacity to absorb new roles. Integration should be coupled with re-design of the HIV M&E system to ensure only a small set of relevant HIV data are collected to address needs at different levels of the health system, and to rationalise data reporting such that health facilities report up the health system hierarchy less frequently and only on a select set of priorities. Most data could rather be incorporated into supervision systems so facility managers and district managers use them for continuous monitoring and service improvement [55]. Inculcating data use to support health management at these levels contributes to South Africa's re-engineering primary health care (PHC) strategy [56].

A few other issues would need to be considered. First, HIV M&E integration entails alignment of both operational and administrative (or managerial) integration [21]. Operational integration would mean integrating HIV data collection and collation forms, data analysis software and templates, and information dissemination mechanisms with those of the overall HIS; and absorbing all HIV M&E-specific personnel (clerks, information officers and managers) within the HIS. It has been shown that the success of any changes to operational processes requires also paying attention to management issues [57]. As such managerial integration would mean assigning responsibility for overseeing the production and use of disease-specific information to district managers (rather than programme managers) and building their M&E skills. Enabling managers at the district level in this manner is a way of promoting integrated health management, especially in decentralised health system settings [58,59].

Second, HIV M&E integration should occur alongside overall HIS strengthening. This undoubtedly requires resources, but some costs may be offset by efficiency gains due to reduced duplication consequent on integration [60]. For HIV M&E integration to maximise programme-system synergies, ear-marked HIV resources could be leveraged and channelled to develop overall HIS capacity to produce more timely and reliable disease-specific and general service data. For example, HIV funding could be used to pay salaries of absorbed HIV M&E personnel, train staff, and improve HIS infrastructure. Lessons can be drawn from integration of HIV laboratory services in Nigeria, whereby using ear-marked HIV funding to train all laboratory personnel and rehabilitate general laboratory infrastructure improved the quality of laboratory performance and benefitted both HIV programme and health system goals [61].

Third, the process of integration should be considered. We propose a phased process guided by operational research, and a bottom-up approach including relevant role players to engender ownership and enhance acceptance of new ways of working [59]. This means recognizing actors at lower levels of the health system and enabling them to take a lead role in their respective jurisdictions without always having to await approval from 'the top'. The fact that health care providers at clinic level in our study developed their own systems suggests that there is problem-solving capacity at sub-district level and this should be tapped, encouraged and supported. It also means getting consensus on actions and timelines, building trust amongst actors, and anticipating and managing any resistance to the inevitable re-distribution of roles because, as experience shows, those who lose responsibilities may try to re-assert their positions by undermining the process [59].

It is well documented in the literature that it is often donor demands that drive vertical reporting of data in low and middle income countries, often placing a heavy data reporting burden on health services. Our study provides a less-documented situation in which national government (funder) demands on sub-national levels (recipients) drive duplicate reporting and undermine data availability and use at district level. Though we used one case study district we expect the findings documented here to be transferable to other district municipalities in South Africa, especially in provinces that operate HIV M&E systems in parallel to the DHIS.

We acknowledge that comprehensive HIV programme integration cannot address the M&E function in isolation. Research is thus needed to understand HIV programme interactions with other health system functions. For a complete picture of programme integration in South Africa, further research should examine how other targeted programmes interact with the health system. Work is also needed to understand interactions and possible tensions between programme and health system managers-an important aspect of integration not addressed in this study [21,62]. We anticipate that the issues highlighted here can inform further studies. Finally, though availability of information at the district level is a necessary first step, we recognise that the purpose of M&E is information use [7] and so recommend further research to understand if and how programme information is used at district level.

## Conclusions

We have highlighted some of the limitations of vertical HIV M&E systems, and some broad factors that my influence their non-integration with the health system. Using a South African case study, we propose integration of the HIV M&E system with the overall health information system (HIS), while concurrently addressing any HIS weaknesses and building programme M&E skills of health system personnel. We view M&E integration as being about more than just merging data recording forms and reporting and dissemination mechanisms. It is about breaking away from historical silo funding that promotes disease-specific M&E systems over strengthening overall health information systems and about building the capacity of HIS personnel and health system managers to take on programme-specific M&E coordination responsibilities. It is an incremental learning process that will require political commitment and investment of time and resources. Leveraging ear-marked HIV funding to provide the needed resources to build overall HIS capacity is a practical way of translating health system strengthening intents of national HIV policy into tangible action.

## Competing interests

The authors declare that they have no competing interests.

## Authors' contributions

MK and SF designed the study methodology. MK carried out the data collection and analysed the data. MK, DB, and SF all contributed to data interpretation and writing, reviewing, and conceptually revising subsequent drafts of the manuscript. All authors read and approved the final manuscript.
